# Database of Amphibia distribution in West Siberia (Russia)

**DOI:** 10.3897/BDJ.10.e82436

**Published:** 2022-04-14

**Authors:** Evgeniy Simonov, Valentina N Kuranova, Artem Lisachov, Vadim V Yartsev, Irina N Bogomolova

**Affiliations:** 1 Severtsov Institute of Ecology and Evolution, Russian Academy of Sciences, Moscow, Russia Severtsov Institute of Ecology and Evolution, Russian Academy of Sciences Moscow Russia; 2 University of Tyumen, Institute of Environmental and Agricultural Biology (X-BIO), Tyumen, Russia University of Tyumen, Institute of Environmental and Agricultural Biology (X-BIO) Tyumen Russia; 3 National Research Tomsk State University, Institute of Biology, Tomsk, Russia National Research Tomsk State University, Institute of Biology Tomsk Russia; 4 Institute of Cytology and Genetics SB RAS, Novosibirsk, Russia Institute of Cytology and Genetics SB RAS Novosibirsk Russia; 5 Siberian State Medical University, Tomsk, Russia Siberian State Medical University Tomsk Russia; 6 Institute of Systematics and Ecology of Animals of the Siberian Branch of the RAS, Novosibirsk, Russia Institute of Systematics and Ecology of Animals of the Siberian Branch of the RAS Novosibirsk Russia

**Keywords:** Anura, batrachofauna, bibliography, Bufonidae, Caudata, digitisation, Hynobiidae, Pelobatidae, Ranidae, Salamandridae, species occurrence

## Abstract

**Background:**

West Siberia is a large region in North Eurasia, which harbours multiple climatic zones, landscape types and biomes. Its amphibian fauna is characterised by a combination of European and Asian species. For many species, this region is the place where the limits of their global ranges are located (*Ranatemporaria*, *R.amurensis*, *Bufotessitibundus*). West Siberia also has at least two non-native amphibian species (*Pelophylaxridibundus*, *Bufotesviridis*). The exact ranges and patterns of distribution of the West Siberian amphibian species are poorly studied. The mapping of species ranges is important for the development of conservation measures and monitoring of invasive species is required to investigate their impacts on the natural ecosystems.

**New information:**

This work presents the most complete biogeographic and occurrence records database of the amphibians of West Siberia. To assemble the database, we digitised data from 190 published works, obtained data from major museum collections and from the data bank on the abundance and distribution of animals «Zoomonitor» by the Zoomonitoring laboratory of the Institute of Systematics and Ecology of Animals, Siberian Branch of Russian Academy of Sciences. The database also includes original and partly unpublished data collected by the authors from 1975 to 2021, as well as quality-assessed citizen science data from the iNaturalist portal. In total, the database contains 2530 records for 11 species of amphibians, including the locality data, the observation date (when known) and the source of the observation (at least one of the following: literature reference, museum sample ID, observer’s name, iNaturalist link).

## Introduction

West Siberia is a vast area encompassing more than 2.4 million km^2^ in North Eurasia and lying between the Ural Mountains and the Yenisei River. West Siberia extends almost 3000 km from north to south and has five ecological zones: tundra, forest-tundra, taiga, forest-steppe, steppe and the Altai-Sayan mountain system with an altitudinal zonation (Suslov 1947). The amphibian fauna of West Siberia is poor due to harsh climatic conditions and includes nine native and two invasive species.

First scarce data on amphibian fauna of West Siberia and adjacent parts of Kazakhstan were obtained in the second half of the 18^th^ century and in the 19^th^ century by such scientists and travellers as J. P. Falck (1824), P. S. Pallas (1831), O. Finsch, A. Brehm (Finsch and Brehm 1882), K. M. Deryugin (1898), V. S. Elpatyevskiy (1901), L. P. Sabaneev (1872), P. Y. Slovtsov (1892), N. F. Kaschenko (1902) and L. S. Berg (1909). More detailed ecological, faunistic and biogeographical studies began in the second part of the 20^th^ century. Historical reviews of herpetological studies in West Siberia are given in the works of V. A. Yakovlev and V. N. Kuranova (Yakovlev 1984, Kuranova 2003, Yakovlev 2011). These works conclude that the distribution of amphibians in West Siberia is still insufficiently studied. The most complete data exist for the Altai Republic (Yakovlev 1999), Tomsk oblast (Kuranova 1998, Kuranova 2000) and for some of the species (*Lissotritonvulgaris* - Skorinov et al. 2008, *Bufotespewzowi* - Litvinchuk et al. 2010). Data on the abundance and distribution of amphibians, based on pitfall trapping, are also available throughout the West Siberian Plain and Altai-Sayan mountain country (Ravkin and Lukyanova 1976, Ravkin et al. 1998, Borisovich et al. 2002, Ravkin et al. 2003, Ravkin et al. 2005, Ravkin et al. 2008, Ravkin et al. 2010). However, large parts of West Siberia (such as administrative regions Omsk Oblast, Yamalo-Nenets and Khanty-Mansi Autonomous Okrugs) remain virtually unexplored. The main reasons behind this lack of information are the vast sizes of the territory, the difficulty to access and sample them and an insufficient number of field herpetologists. The aim of this study is to summarise all possible sources of occurrence records for amphibians in West Siberia, including our unpublished data, published literature records and verified reports on “iNaturalist”.

### Overview of the amphibian species of West Siberia

Amongst the 11 species recorded in West Siberia, widely distributed species are the Siberian salamander *Salamandrellakeyserlingii* Dybowski, 1870, the common toad *Bufobufo* (Linnaeus, 1758), the moor frog *Ranaarvalis* Nilsson, 1842 and the Siberian wood frog *R.amurensis* Boulenger, 1886. The eastern and north-eastern periphery of the ranges of the common newt *Lissotritonvulgaris* (Linnaeus, 1758), the Pallas’ spadefoot toad *Pelobatesvespertinus* (Pallas, 1771), the variable toad *Bufotessitibundus* (Pallas, 1771), Pewzow’s toad *Bufotespewzowi* (Bedriaga, 1898), the common frog *R.temporaria* Linnaeus, 1758 and the frogs of *Pelophylaxridibundus* (Pallas, 1771) complex are located in the region. The green toad *Bufotesviridis* (Laurenti, 1768) exists in West Siberia outside its native range, as an inadvertently introduced species. Below, we provide a brief description of the distribution, habitats and regional conservation status of all amphibian species inhabiting West Siberia.

*Salamandrellakeyserlingii* Dybowski, 1870

The Siberian salamander is the amphibian species with the largest native range in the world (Poyarkov and Kuzmin 2008). Its range covers the European part of Russia, the Urals and Siberia and spans to the Chukotka and Kamchatka Peninsulas in the east. In the south, the range of the Siberian salamander reaches north Kazakhstan, north China and north Mongolia (Kuzmin 2012). In West Siberia, this species mainly inhabits the taiga zone, entering the tundra zone in the north and the steppe zone in the south through the forested river valleys. In the southeast of the forested areas of West Siberia, the Siberian salamander inhabits birch-aspen, birch-fir and birch-pine forests and swamps. It is common in raised bog areas in the taiga zone (Ravkin et al. 1998, Ravkin et al. 2005, Ravkin et al. 2008). The salamanders typically breed in the water bodies situated in the margins of the raised bog areas in pine forests, in shallow overgrown lakes and temporary water bodies in the interfluves, as well as in roadside and melioration ditches (Kuzmin 2012). At the same time, the salamanders do not inhabit large rivers like Ob, Tom, Ket and their tributaries and large and small water bodies situated in their floodplains (Ravkin et al. 2005, Ravkin et al. 2008).

We report 290 locations where this species was observed in West Siberia, from 67.33°N to 52.04°N in latitude and throughout the whole region longitudinally.

The Siberian salamander is listed in the Red Books of Kurgan Oblast (category III, rare species) (Bolshakov et al. 2012), Omsk Oblast (category III, rare species, under the name “*Hynobiuskeyserlingi*”) (Sidorov and Plikina 2015), Altai Krai (category IV, data deficient) (Irisova et al. 2016), Chelyabinsk Oblast (category II, vulnerable) (Bolshakov 2017), Yamalo-Nenets Autonomous Okrug (category III, rare species) (Ektova and Zamyatin 2010) and Sverdlovsk Oblast (category IV, data deficient) (Korytin 2018).

*Lissotritonvulgaris* (Linneus, 1758)

The territory of West Siberia is inhabited by the nominative subspecies, *Lissotritonvulgarisvulgaris* (*Kuzmin 2012*). The majority of the known localities in West Siberia are situated at the border between the forest and forest-steppe zones, where the newts occur in birch-aspen and pine forests. In the valleys of large rivers, the newts prefer forested hills, which are not flooded during the spring floods. In the forest-steppe, the density of the newt populations decreases to the south and south-east with the climate aridisation (Kuzmin 2012). Another limitation of the occurrence of common newts is the presence of water bodies suitable for reproduction. The newts prefer open and well-heated water bodies in forested areas and in river floodplains (Skorinov et al. 2008).

We report 176 registrations. The known northern limit of the distribution is declining to the south when moving to the east (from 62.0°N in Khanty-Mansi Autonomous Okrug to 56.5°N in Kemerovo Oblast). The southern distribution limit follows the transition zone between forest-steppe and steppe zones (from 54.7°N on the west to 51.1°N on the east). In the east, the distribution is limited by the Altai and Kuznetsk Alatau mountains. The easternmost known location is situated in Krasnoyarsk Krai (56.53°N 89.32°E).

The common newt is listed in the Red Books of Tyumen Oblast (category IV, data deficient) (Petrova 2020), Omsk Oblast (category III, rare species, under the name “*Triturusvulgaris*”) (Sidorov and Plikina 2015), Kemerovo Oblast (category I, endangered) (Skalon 2012), Krasnoyarsk Krai (category IV, data deficient) (Savchenko 2012), Tomsk Oblast (category VI, “a species of aesthetic and scientific value”) (Adam 2013) and Khanty-Mansi Autonomous Okrug (category IV, data deficient) (Vasin and Vasina 2013).

*Ranaamurensis* Boulenger, 1886

The Siberian wood frog is an Asiatic species, which inhabits West and East Siberia, the Far East, North Korea, Manchuria, northern and central Mongolia. Several populations are known north of the Arctic Circle. The southern border of its range runs through southern Siberia to Mongolia, Manchuria and Korea (Kuzmin 2012). The south-western border of the range is situated in Kurgan Oblast. Here, the frog is found in the north-eastern part of the region, where it is confined to the floodplains of rivers and near lake shores (Starikov 2014). It inhabits altitudes of 0–500 m above sea level (Kuzmin 2012). The presence and hydrochemical characteristics of water bodies suitable for wintering is an important factor for the species. In this regard, over most of the range, especially on its periphery, the Siberian wood frog is associated with river floodplains (Kuzmin 2012). On the territory of West Siberia, this species is found from the middle taiga to the southern forest-steppe. In the middle and southern taiga, it prefers floodplain swamps and, to a lesser extent, meadows. The abundance of the species is maximal in the southern taiga (Ravkin et al. 1998).

We registered 115 records of the Siberian wood frog. The western border of the species range goes through the 63-62°E longitude. The northernmost record of the species in West Siberia is at 65.55°N.

The Siberian wood frog is listed in the Red Book of Kurgan Oblast (category III, rare species) (Bolshakov et al. 2012), Kemerovo Oblast (category III, rare species) (Skalon 2012), Krasnoyarsk Krai (category III, rare species) (Savchenko 2012), Khanty-Mansi Autonomous Okrug (category III, rare species) (Vasin and Vasina 2013), Yamalo-Nenets Autonomous Okrug (category IV, data deficient) (Ektova and Zamyatin 2010) and Sverdlovsk Oblast (category III, rare species) (Korytin 2018).

*Ranaarvalis* Nilsson, 1842

The moor frog is distributed from Western Europe (Germany, Belgium, Sweden) to East Siberia (Republics of Sakha and Buryatia (Russia)). The southern limit of the range lies in the steppe regions of north Kazakhstan and Mongolia and in the Altai mountains in Russia, north China and Mongolia (Kuzmin 2012). The moor frog inhabits diverse habitats, such as swamps, forests, meadows and river floodplains. In the tundra and in the steppe, the moor frog prefers forested or bush areas in the river valleys, as well as vicinities of other water bodies (Ravkin et al. 2005, Ravkin et al. 2008). It frequently inhabits anthropogenic habitats, including gardens and city parks (Kuzmin 2012).

In West Siberia, the moor frog is the most common amphibian species, which is present throughout the whole studied region, except the furthest north (Yamal and Gyda Peninsulas) and the highest areas of Altai Mountains. The species crosses the Arctic Circle and is distributed up to the Village Tazovskiy (67.47°N; the northernmost record of Amphibia in West Siberia). Here, we report 1137 records of the species.

Due to its abundance, the moor frog is not included in Red Data books in any of the administrative regions of West Siberia.

*Ranatemporaria* Linnaeus, 1758

The common frog is distributed from the Pyrenees to the Urals and West Siberia. The north-eastern border of its range runs from the southern coast of the Barents Sea and the northern coast of the White Sea to southeast and east through the Komi Republic to the Rivers Ob and Irtysh. The south-eastern border of the range is situated in the Kurgan Oblast and north Kazakhstan (Kuzmin 2012). In the northern and southern peripheries of its range, the species is usually found in the vicinity of water bodies (Ravkin et al. 2005, Ravkin et al. 2008).

We report 40 records of this species in West Siberia, from 67.0°N in the north to 55.43°N in the south and to 67.08°E in the east. The eastern border of the range in West Siberia is insufficiently known. In the Yamalo-Nenets Autonomous Okrug, only three populations are known, although there are more finds in the Khanty-Mansi Autonomous Okrug, where the common frog inhabits western and north-western parts of the region.

The common frog is listed in the Red Books of Tyumen and Kurgan Oblasts, Yamalo-Nenets and Khanty-Mansi Autonomous Okrugs as a rare species (category III) (Bolshakov et al. 2012, Vasin and Vasina 2013, Petrova 2020).

*Pelophylaxridibundus* (Pallas, 1771) complex

The frogs of *P.ridibundus* complex have a disjunct distribution in West Siberia due to its unintended introduction to some areas. The natural range of this group runs from Eastern Europe to the extreme southwest of Siberia, surrounding the Ural Mountains from the south, since these frogs do not inhabit the forested montane areas of the Urals (Kuzmin 2012, Fominykh et al. 2016).

In West Siberia, the western populations occur in Chelyabinsk Oblast, Sverdlovsk Oblast and Kurgan Oblast. Their origin is debatable and could represent natural range extension in the second half of the 20^th^ century, spread of invasive populations or combination of both (Fominykh et al. 2016). The eastern, purely invasive populations occur in Omsk Oblast, Tomsk Oblast, Novosibirsk Oblast, Kemerovo Oblast, Altai Krai, Altai Republic, Republic of Khakassia and Krasnoyarsk Krai. The natural sources of introduced populations are hitherto unknown and it is possible that the region is inhabited by several species of the complex and their hybrids. We suppose the presence of *P.ridibundus* sensu stricto and P.cf.bedriagae, which are widely distributed in the European part of Russia (Litvinchuk et al. 2020). Overall, we report 170 records of *Pelophylaxridibundus* sensu lato in West Siberia.

*Bufobufo* (Linnaeus, 1758)

The common toad is widely distributed in Eurasia, including West and East Siberia. This species mainly inhabits forested areas and prefers swamped conifer forests. It is also encountered in mixed and leaf forests, groves, parks and gardens, where high humidity and dense vegetation are present (Kuzmin 2012). The common toad is less abundant in north taiga than in middle taiga, where it is most numerous in fir, spruce, birch and aspen forests. In south taiga, the toad prefers lowland swamps. In sub-taiga, it is most abundant in swamps and swamped forests, but is also common in drier habitats. In the north forest-steppe, it inhabits birch and aspen forests and forest patches surrounded by agricultural fields. In the south forest-steppe, which is the southern border of its range, the common toad is very rare and inhabits only lowland swamps. This species is not found in the steppes (Ravkin et al. 1998, Ravkin et al. 2003, Ravkin et al. 2005, Ravkin et al. 2008).

We report 539 localities where the common toad occurs. The northern periphery of its range lies between 63.0°N and 64.0°N. The southern range margin in West Siberia is situated in Kurgan Oblast (Yurgamyshskiy and Ketovskiy Districts, at 55.0°N), then it runs through the south of Tyumen Oblast and east of Omsk Oblast to north-eastern Kazakhstan.

The common toad is listed in Red Data Books of Kurgan Oblast (category IV, data deficient) (Bolshakov et al. 2012), Omsk Oblast (category III, rare species) (Sidorov and Plikina 2015) and Yamalo-Nenets Autonomous Okrug (category III, rare) (Ektova and Zamyatin 2010).

*Bufotesviridis* (Laurenti, 1768)

An isolated, apparently introduced population of green toads occurs in the vicinity of the city of Novosibirsk. In 1984, an established population of green toads already existed near Novosibirsk (Zolotarenko 1985). Genome size analyses of three toads and microsatellite genotyping of one toad from this population suggested that they belong to the species *B.sitibundus*, the variable toad, which naturally occurs in Eastern Europe east of the Volga River, in the Urals, Caucasus and parts of Central Asia (Litvinchuk et al. 2007). However, a recent analysis of genome size and RAD-Seq genotyping of another sample showed that it belongs to *B.viridis* sensu stricto, which does not inhabit Central Asia (Dufresnes et al. 2019). Thus, the vicinity of Novosibirsk is apparently inhabited by two species of green toads. More sampling data are required to clarify their distributions and determine the source or sources of the introduction. Near Novosibirsk, the green toads inhabit pine forests (the Novosibirsk Zoo and the park around it), agricultural landscapes with patches of birch forest (near the Village Verkh-Tula) and other habitats.

*Bufotessitibundus* (Pallas, 1771)

The variable toad occurs in south-western parts of West Siberia. We report 26 records of this toad in West Siberia, all in the Chelyabinsk and Kurgan Oblasts. The northernmost location is at 56.21°N and the easternmost location lies at 65.45°E. The occurrence of this species is also possible in the Altai Krai, although there is currently no strong evidence for it.

Under the name “*Pseudepidaleaviridis*”, it is listed in the Red Data Book of Kurgan Oblast (category III, rare species) (Bolshakov et al. 2012).

*Bufotespewzowi* (Bedriaga, 1898)

Historically, the green toads from the montane regions of the Altai Republic were presumed to belong to *B.viridis*. In 2010, the analysis of chromosome number and genome size revealed that they belong to the tetraploid species *B.pewzowi* (Litvinchuk et al. 2010), which is widely distributed in Kazakhstan, Mongolia and China. This was the first report of this species from Russia. In general, there are nine known localities, situated from 51.12°N to 49.65°N and from 500 m a.s.l. to 1700 m a.s.l.

The species is listed in the Red Book of Altai Republic (category III, rare species) (Bondarenko 2017) and in the Red Book of Russia (category 2, EN) (Pavlov 2021).

*Pelobatesvespertinus* (Pallas, 1771)

The Pallas’ spadefoot toad is distributed from Eastern Europe and Caucasus to West Siberia and Kazakhstan (Kuzmin 2012). At the eastern periphery of the range, the spadefoot toad is most numerous in sub-taiga forests, mainly in pine forests on sandy substrates. In the northern forest-steppe zone, the toad mostly occurs in open swamp landscapes. In the southern forest-steppe, it prefers forest patches near fields and meadows. In the steppe, it occurs mainly in halophyte meadows and flood-meadows and, more rarely, in swamp areas around freshwater lakes (Ravkin et al. 2008).

We report 32 records of this species in West Siberia. The north-eastern limit of its range is situated in the Tyumen Oblast (near the City Tobolsk, 58.17°N, 68.33°E).

The spadefoot toad is listed under the name “*P.fuscus*” in the Red Books of Tyumen and Sverdlovsk Oblasts as a rare species (category III) (Korytin 2018, Petrova 2020).

## Project description

### Title

Database of Amphibia distribution in West Siberia (Russia)

### Personnel

Evgeniy Simonov, Valentina Kuranova, Artem Lisachev, Vadim Yartsev, Irina Bogomolova

### Study area description

The designated study area is limited on the south by the administrative border of the Russian Federation, by the Arctic Ocean shore on the north, by foothills of the eastern slope of Ural Mountains on the west and by the Yenisey River and administrative border of Tuva Republic on the East. The area extends up to about 1890 km from the west to the east and from north to south up to 2800 km. The total area equals about 2.4 million km^2^ and covers a number of zones (Suslov 1947). The tundra zone occupies the extreme north of the West Siberian Plain. There are three tundra subzones: arctic, typical (moss-lichen) and southern (shrubs). Forest-tundra is located to the south of the tundra and up to 150 km wide. It is a transition zone, so it is covered with areas of sparse forests, bogs and shrubs. The forest zone (taiga) covers the area between 66° and 56° N and is about 1000 km wide. The forest zone of the West Siberian Plain is divided into sub-zones of northern, middle, southern taiga and birch-aspen forests. The forest-steppe zone is located to the south of the taiga and characterised by the presence of both forest and steppe plant communities, as well as marshes, salt marshes and meadows. Another characteristic feature of the forest-steppe of West Siberia is the abundance of saline drainless lakes. The steppe zone covers the southern part of the Omsk Oblast and the south-western part of the Novosibirsk Oblast, as well as the western part of Altai Krai. In the Altai-Sayan mountain system, including relatively low ridges like Salair and Kuznetsk Alatau, altitudinal zonation is present. The steppes and forest steppes at the foothills of these mountains are replaced with montane forests in the higher altitude areas and alpine meadows and montane semi-deserts in the highest areas of Altai.

## Sampling methods

### Study extent

Original field surveys data, available scientific literature, museum collections, personal reports and iNaturalist (https://www.inaturalist.org) observations were used to arrange the database. The species occurrence records were obtained from 190 publications. Data from specimens stored in the herpetological collections of the following museums were used: Zoological Museum of Moscow State University (ZMMU), Zoological Museum of Tomsk State University (TSU), Zoological Museum of Institute of Systematics and Ecology of Animals and Siberian Branch of Russian Academy of Sciences (ISEA). We also used data from the «Zoomonitor» database by the Zoomonitoring laboratory of Institute of Systematics and Ecology of Animals, Siberian Branch of RAS (http://eco.nsc.ru/zoomonit/zoomonit_r.htm). The geographical extent fully covered nine administrative regions of Russia (Kemerovo, Kurgan, Omsk, Novosibirsk, Tomsk and Tyumen Oblasts, Altai Krai, Altai and Khakasia Republics); almost fully, except for the most western parts - Khanty-Mansi and Yamalo-Nenets Autonomous Okrugs; eastern parts of Chelyabinsk and Sverdlovsk Oblasts and the western part of Krasnoyarsk Krai.

### Sampling description

We collected the species occurrence data during field studies in Siberia in 1975-2021. The direct visual observations of animals were used most often, but in some cases, pitfall traps were employed. A portion of our data has been published earlier, but scattered in various publications and not always had a geographical coordinate reference. The majority of amphibia species records from publications were also made by direct observations.

### Quality control

The majority of data (including published literature) were collected by herpetologists. Personal communications from other researchers or amateur naturalists were included if accompanied by photos. For iNaturalist, we exported all “Research grade” observations of amphibians from the areas of interest. Then, the observations were manually checked and wrong species identifications were corrected. If the quality of photographs did not allow us to check the correctness of species identification, such observations were removed from our dataset. For each observation, we manually added the locality data (stateProvince, county and locality fields), based on the geographic coordinates, if these data were not determined automatically or not provided by authors of the observations. Incorrect or obsolete species names were corrected when adding records to the database. The modern names of administrative units were used. The compiled database went through several rounds of revision in a search of mistypes and other kinds of errors.

### Step description

The occurrence records from literature or our own old data were georeferenced using Yandex (https://yandex.ru/maps) or Google (https://maps.google.ru/maps) maps services from verbal depictions of localities or using coordinates provided in these publications by authors. The same procedure was applied for the records from the museums’ collections.

Geographical coordinates for our own observations have been determined using hand-held GPS units (accuracy: 11 m) since 2007 in most cases.

The locality names reported in Russian were transliterated into English.

The statistical and spatial analyses of the final database were made using the Plotly library for Python and QGIS 3.16. Species richness map was created using FSC QGIS Plugin for biological recorders by aggregating records in a 40 km squares.

Bibliographic references were assembled in the original language and English translation; web-links to full texts or abstracts are provided when possible (Suppl. material 1).

## Geographic coverage

### Description

The study area is limited on the south by the administrative border of the Russian Federation, by the Arctic Ocean shore on the north, by foothills of eastern slope of Ural Mountains on the west and by the Yenisey River and administrative border of Tuva Republic on the east. The area extends up to about 1890 km from the west to the east and from north to south up to 2800 km. The total area equals about 2.4 million km^2^.

**Figure 1.**
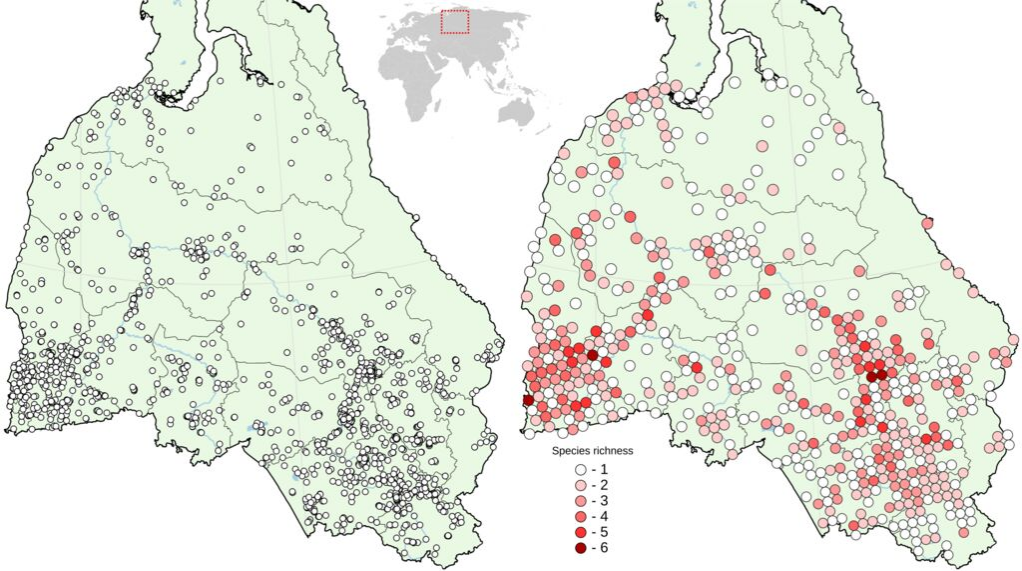
The distribution of the occurrence records included in the database (left) and species richness (right) in West Siberia.

Amphibian records data have a very uneven distribution, resulting in a species richness map better reflecting ‘sampling effort’ than the real biogeographical pattern (Fig. 1). More than half of the records (51.6%) belong to the three most studied administrative regions - Tomsk Oblast (18.2%), Novosibirsk Oblast (17.9%), and Altai Krai (15.5%). Nevertheless, the number of species encountered, averaged over the zones as a whole, clearly reflects a decrease in species richness from north to south, from zero in the Arctic and northern subarctic tundras to the forest-steppe, followed by a decrease in the steppe zone. From east to west, the largest number of species was observed in the south-western part due to the presence of the European species. In the Altai-Sayan mountain system, the species richness decreases with an increase in absolute altitude.

### Coordinates

49.62 and 67.47 Latitude; 60.32 and 93.40 Longitude.

## Taxonomic coverage

### Description

**Figure 2.**
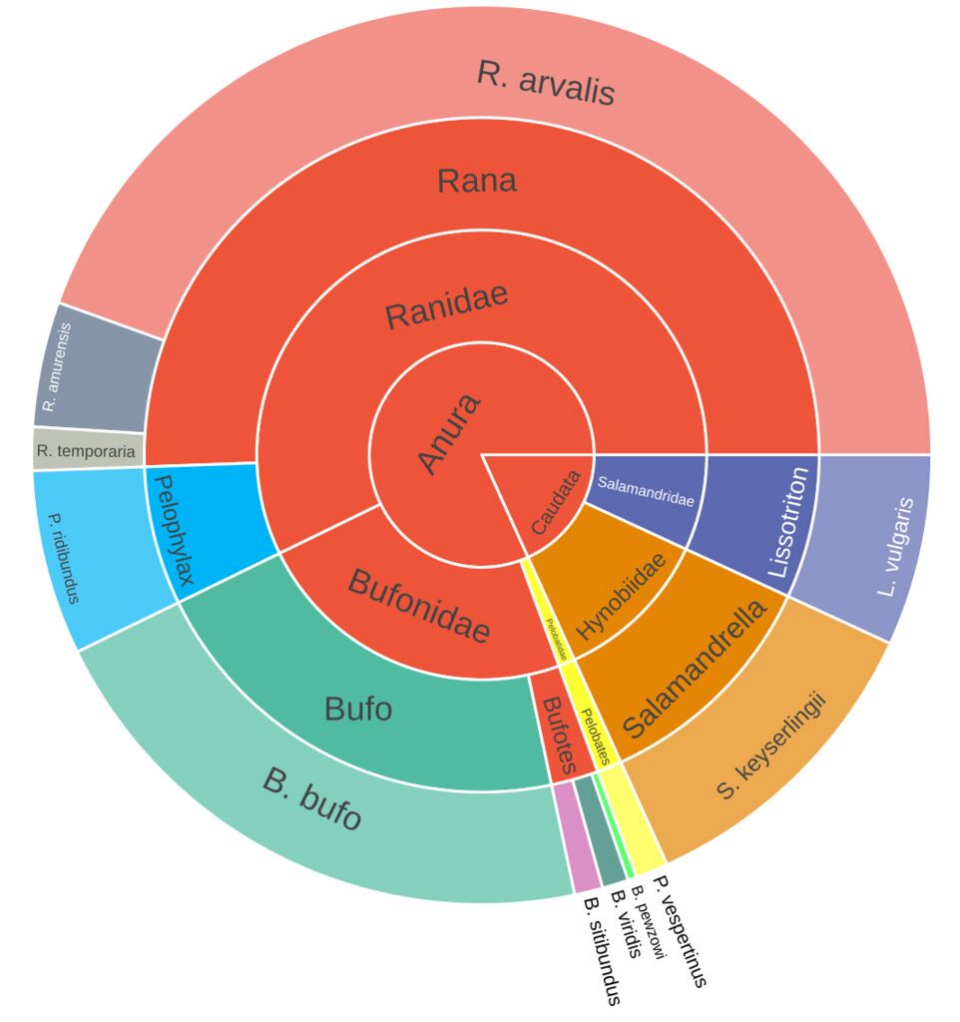
Taxonomic distribution of amphibia species occurrence records in the dataset.

The database contains records for 11 species, representing seven genera, five families and two orders of Amphibia (Fig. 2). Taxonomy follows “Amphibian Species of the World: an online reference. Version 6.1” (Frost 2021).

### Taxa included

**Table:** 

Rank	Scientific Name	Common Name
phylum	Chordata	chordates
class	Amphibia	amphibians
order	Caudata	salamanders
order	Anura	frogs
family	Hynobiidae	Asiatic salamanders
family	Salamandridae	True salamanders
family	Pelobatidae	European spadefoot toads
family	Bufonidae	true toads
family	Ranidae	true frogs
species	*Salamandrellakeyserlingii* Dybowski, 1870	Siberian salamander
species	*Lissotritonvulgaris* (Linneus, 1758)	common newt
species	*Pelobatesvespertinus* (Pallas, 1771)	Pallas’ spadefoot toad
species	*Bufotessitibundus* (Pallas, 1771)	variable toad
species	*Bufotesviridis* (Laurenti, 1768)	green toad
species	*Bufotespewzowi* (Bedriaga, 1898)	Pewzow’s toad
species	*Bufobufo* (Linnaeus, 1758)	common toad
species	*Ranaarvalis* Nilsson, 1842	moor frog
species	*Ranaamurensis* Boulenger, 1886	Siberian wood frog
species	*Ranatemporaria* Linnaeus, 1758	common frog
species	*Pelophylaxridibundus* (Pallas, 1771)	marsh frog

## Temporal coverage

**Data range:** 1856-1-01 – 2021-8-01.

## Usage licence

### Usage licence

Other

### IP rights notes

This work is licensed under a Creative Commons Attribution (CC-BY) 4.0 License.

## Data resources

### Data package title

Database of Amphibia distribution in West Siberia (Russia).

### Resource link

https://www.gbif.org/ru/dataset/7027c22a-dca9-49db-8f5a-93c3ccc6072b

### Alternative identifiers

https://doi.org/10.15468/44nx2c

### Number of data sets

1

### Data set 1.

#### Data set name

Database of Amphibia distribution in West Siberia (Russia).

#### Data format

Darwin Core

#### Number of columns

21

#### Download URL

https://www.gbif.org/ru/dataset/7027c22a-dca9-49db-8f5a-93c3ccc6072b

#### Description

The dataset includes a table in Darwin Core format with 21 fields and 2530 records. The earliest first record dates back to 1856 and the most recent event occurred in 2021 (Simonov et al. 2021).

**Data set 1.** 

Column label	Column description
occurrenceID	An identifier for the Occurrence (as opposed to a particular digital record of the occurrence). In the absence of a persistent global unique identifier, construct one from a combination of identifiers in the record that will most closely make the occurrenceID globally unique.
order	The full scientific name of the order in which the taxon is classified.
family	The full scientific name of the family in which the taxon is classified.
genus	The full scientific name of the genus in which the taxon is classified.
scientificName	The full scientific name, with authorship and date information, if known. When forming part of an Identification, this should be the name in lowest level taxonomic rank that can be determined. This term should not contain identification qualifications, which should instead be supplied in the IdentificationQualifier term.
country	The name of the country or major administrative unit in which the Location occurs.
stateProvince	The name of the next smaller administrative region than country (state, province, canton, department, region etc.) in which the Location occurs.
county	The full, unabbreviated name of the next smaller administrative region than stateProvince (county, shire, department etc.) in which the Location occurs.
locality	The specific description of the place. Less specific geographic information can be provided in other geographic terms (higherGeography, continent, country, stateProvince, county, municipality, waterBody, island, islandGroup). This term may contain information modified from the original to correct perceived errors or standardise the description.
verbatimCoordinates	The verbatim original spatial coordinates of the Location. The coordinate ellipsoid, geodeticDatum or full Spatial Reference System (SRS) for these coordinates should be stored in verbatimSRS and the coordinate system should be stored in verbatimCoordinateSystem.
verbatimCoordinateSystem	The coordinate format for the verbatimLatitude and verbatimLongitude or the verbatimCoordinates of the Location.
decimalLongitude	The geographic longitude (in decimal degrees, using the spatial reference system given in geodeticDatum) of the geographic centre of a Location. Positive values are east of the Greenwich Meridian, negative values are west of it. Legal values lie between -180 and 180, inclusive.
decimalLatitude	The geographic latitude (in decimal degrees, using the spatial reference system given in geodeticDatum) of the geographic centre of a Location. Positive values are north of the Equator, negative values are south of it. Legal values lie between -90 and 90, inclusive.
coordinateUncertaintyInMetres	The horizontal distance (in metres) from the given decimalLatitude and decimalLongitude describing the smallest circle containing the whole of the Location.
geodeticDatum	The ellipsoid, geodetic datum or spatial reference system (SRS) upon which the geographic coordinates given in decimalLatitude and decimalLongitude are based.
eventDate	The date-time or interval during which an Event occurred. For occurrences, this is the date-time when the event was recorded. Not suitable for a time in a geological context.
basisOfRecord	The specific nature of the data record.
recordedBy	A list (concatenated and separated) of names of people, groups or organisations responsible for recording the original Occurrence. The primary collector or observer, especially one who applies a personal identifier (recordNumber), should be listed first.
associatedReferences	A list (concatenated and separated) of identifiers (publication, bibliographic reference, global unique identifier, URI) of literature associated with the Occurrence.
institutionCode	The name (or acronym) in use by the institution having custody of the object(s) or information referred to in the record.
materialSampleID	An identifier for the MaterialSample (as opposed to a particular digital record of the material sample). In the absence of a persistent global unique identifier, construct one from a combination of identifiers in the record that will most closely make the materialSampleID globally unique.

## Supplementary Material

F4B399E7-0827-5092-A1F3-C9C87BE2BADE 10.3897/BDJ.10.e82436.suppl1 Supplementary material 1 Bibliography of research containing occurrence records Data type Bibliography Brief description The bibliography of research containing occurrence records for Amphibia in West Siberia. File: oo_645453.txt https://binary.pensoft.net/file/645453 Evgeniy Simonov, Valentina Kuranova, Artem Lisachov, Vadim Yartsev, Irina Bogomolova
